# Mixed Exposure of Persistent Organic Pollutants Alters Oxidative Stress Markers and Mitochondrial Function in the Tail of Zebrafish Depending on Sex

**DOI:** 10.3390/ijerph18189539

**Published:** 2021-09-10

**Authors:** Songhee Lee, Eun Ko, Hyojin Lee, Ki-Tae Kim, Moonsung Choi, Sooim Shin

**Affiliations:** 1 Interdisciplinary Program of Bioenergy and Biomaterials Graduate School, College of Engineering, Chonnam National University, Gwangju 61186, Korea; sohs200@naver.com; 2 Department of Biotechnology and Bioengineering, College of Engineering, Chonnam National University, Gwangju 61186, Korea; ge6008@gmail.com; 3 Department of Environmental Energy Engineering, College of Energy and Biotechnology, Seoul National University of Science and Technology, Seoul 01811, Korea; hyojin_lee@seoultech.ac.kr (H.L.); ktkim@seoultech.ac.kr (K.-T.K.); 4 Department of Optometry, College of Energy and Biotechnology, Seoul National University of Science and Technology, Seoul 01811, Korea; 5 Convergence Institute of Biomaterials and Bioengineering, Seoul National University of Science and Technology, Seoul 01811, Korea

**Keywords:** Aroclor 1254, endocrine disruptors, mitochondrial dysfunction, organochlorine pesticides, oxidative stress, persistent organic pollutants, polychlorinated biphenyls

## Abstract

Persistent organic pollutants (POPs) are lipid-soluble toxins that are not easily degraded; therefore, they accumulate in the environment and the human body. Several studies have indicated a correlation between POPs and metabolic diseases; however, their effects on mitochondria as a central organelle in cellular metabolism and the usage of mitochondria as functional markers for metabolic disease are barely understood. In this study, a zebrafish model system was exposed to two subclasses of POPs, organochlorine pesticides (OCPs) and polychlorinated biphenyls (PCBs), under two different conditions (solitary OCPs or OCPs with PCBs (Aroclor 1254)), and changes in the oxidative stress marker levels and mitochondrial enzyme activities in the electron transport chain of the tail were measured to observe the correlation between POPs and representative biomarkers for metabolic disease. The results indicated different responses upon exposure to OCPs and OCPs with Aroclor 1254, and accelerated toxicity was observed following exposure to mixed POPs (OCPs with Aroclor 1254). Males were more sensitive to changes in the levels of oxidative stress markers induced by POP exposure, whereas females were more susceptible to the toxic effects of POPs on the levels of mitochondrial activity markers. These results demonstrate that the study reflects real environmental conditions, with low-dose and multiple-toxin exposure for a long period, and that POPs alter major mitochondrial enzymes’ functions with an imbalance of redox homeostasis in a sex-dependent manner.

## 1. Introduction

Environmental pollution induced by anthropogenic chemicals is a seriously threatening factor for ecological systems. Several pesticides, insecticides, and herbicides classified as persistent organic pollutants (POPs) were synthesized in the 1900s for various industrial applications; most of them are now prohibited by the Stockholm convention for their persistence in the environment and their associated adverse effects [1]. Among several subclasses of POPs, organochlorine pesticides (OCPs) are chemicals containing at least one covalently bonded atom of chlorine. They also have the characteristics of high lipid solubility and further bioaccumulation in adipose tissues [2,3]. Previous studies have shown that exposure to OCPs is correlated with metabolic diseases (e.g., type 2 diabetes) [4,5,6]. It has been reported that elevated levels of OCPs or low-dose OCPs with long exposure times are associated with an increased risk of diabetes [7,8]. Aroclor 1254 is one of the polychlorinated biphenyls (PCBs) involved with POPs. One major characteristic of PCBs, used to differentiate them from OCPs, is their role as endocrine disruptors, activating androgen transcriptional activity via AhR-AR-Jarid1b interaction and disrupting thyroid and reproductive function [9,10,11].

In the context of the correlation of POPs with metabolic disease and endocrine control, mitochondrial function is considered to have significance in both processes. As a central organelle for energy homeostasis, mitochondria produce cellular energy (ATP) through the process of oxidative phosphorylation, comprising the tricarboxylic acid cycle and the electron transport chain. In addition to energy metabolism imbalance, mitochondrial dysfunction is closely connected to the development of endocrine diseases, since mitochondria also play an important role in cholesterol and hormone biosynthesis [12]. Along with the fundamental function of mitochondria that manipulate metabolic homeostasis in the cell, the mitochondria is one of the sources for the generation of reactive species that serve as secondary messengers to mediate redox signaling [13]. However, alterations in mitochondrial function caused by factors such as mtDNA mutations and environmental toxins induce dysfunction in specific oxidative phosphorylation processes, resulting in energy metabolism imbalances, the overproduction of oxygen radicals that induce oxidative stress, and the further onset of metabolic diseases [14]. To compromise for the harmful effects of reactive species, antioxidant systems such as superoxide dismutase (SOD), catalase (CAT), and reduced glutathione (GSH) function to scavenge these radical species [15]. Although the relationship between POPs and metabolic disease has been examined in several epidemiological studies and some experimental models as possible obesogenic and diabetogenic agents, the disruption of metabolic function following POP exposure and its further effects on mitochondrial function have not been comprehensively studied [16,17,18].

In accordance with the most common method used for investigating exposure to POPs in water, zebrafish were used in the present study. They were also chosen for their advantages of genetic similarity, rapid growth, and their ease of manipulation as an animal model [19,20]. The tail of the zebrafish represents a skeletal muscle, which is a target organ for metabolic disease studies. Moreover, zebrafish have subcutaneous adipose tissue surrounding the tail that facilitates the depositing of lipophilic toxins around the tissue [21].

**Figure 1:**
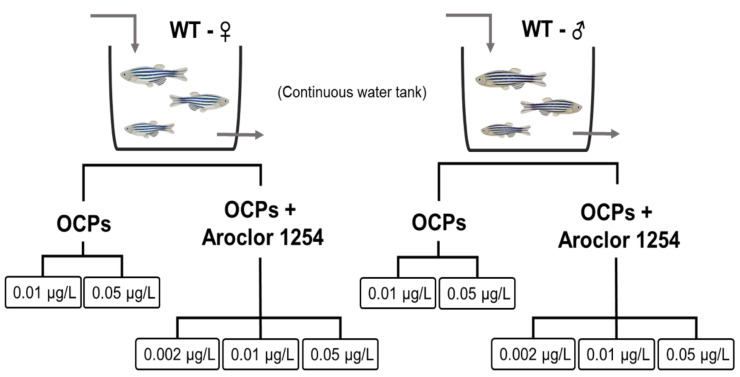
Overall scheme of OCPs or OCPs and Aroclor 1254 exposure.

In the current study, adult zebrafish were exposed to low doses of POPs (solitary OCPs or OCPs with Aroclor 1254 for three months) to identify the metabolic and mitochondrial functional changes caused by POPs. The exposed groups were subdivided according to sex and the target chemicals (Figure 1). Based on the fact that OCPs exist as a mixture, five types of OCPs were selected based on their prevalence in the human body [22,23]. To examine the synergetic effects of the different subclasses of POPs, Aroclor 1254 was mixed with five OCPs. After exposure, the levels of oxidative stress markers and mitochondrial enzyme activities in oxidative phosphorylation were measured in the tail of the zebrafish. The results were compared according to sex and to OCPs or OCPs with Aroclor 1254 to examine the effects of POPs on mitochondrial function in relation to metabolic disease.

## 2. Materials and Methods

### 2.1. Animals and POP Exposure

The adult zebrafish (*Danio rerio*, AB line) were bred in the Korea Testing and Research Institute (Hwasun, Republic of Korea). As for their breeding conditions, 10- to 12-month-old zebrafish were raised in a 14:10 h light:dark cycle at 28 °C. Exposure to the POP mixtures lasted for 12 weeks in a continuous water flow tank through the mixing of a constant volume of stock solution to maintain the POP concentrations. Each day, 0.15 g of Gemma micro was fed three times. Zebrafish were divided into two groups. One was exposed to a mixture of five OCPs (4,4′-DDT, chlordane, heptachlor, beta-HCH, and hexachlorobenzene, mixed in a 1:1:1:1:1 ratio), whereas the other was exposed to Aroclor 1254 in addition to the OCP mixture, mixed at a 1:1 ratio. The concentrations of individual OCPs were set at 0.01 and 0.05 μg/L and the individual concentrations of five OCPs with Aroclor 1254 were set at 0.002, 0.01, and 0.05 μg/L for each chemical. The control groups were only exposed to 0.1% dimethyl sulfoxide, a solvent used to dissolve POPs. A total of 30 zebrafish, including 15 males and 15 females, were used for each concentration batch. The three batches per group were cultivated under each concentration condition for three months in 6 L water tanks, separating the groups according to sex.

### 2.2. Isolation of Mitochondria and Cytosolic Fraction

Zebrafish were dissected after 8–10 h of fasting. Three to six tails were collected with a uniform weight per batch. For the isolation of mitochondria, 300 mg of tissue was stabilized using 500 μL of phosphate-buffered saline (PBS; 100 mM sodium phosphate and 150 mM sodium chloride, pH 7.2) and homogenized using a MagNA Lyser Instrument (Roche, Basel, Switzerland). A mitochondria isolation kit for tissue (Thermo Fisher Scientific, Waltham, MA, USA) was used to separate the mitochondrial proteins from the tails. First, the sample was mixed with 800 μL of bovine serum albumin (BSA)/reagent A to stabilize the sample. Next, 10 μL of reagent B was added and vortexed strongly to disrupt the cells. Finally, 800 μL of reagent C was added and centrifuged at 700× *g* to remove the pellet containing cellular debris. The supernatant was centrifuged at 3000× *g* to separate the mitochondrial protein and the cytosolic fraction. The cytosolic fraction was stored separately, and the mitochondrial protein was washed using a washing buffer. Next, the mitochondrial fraction was resuspended in 100 μL of PBS and stored at −80 °C.

### 2.3. Total Reactive Oxygen Species (ROS)/Reactive Nitrogen Species (RNS) Levels

The total free radical presence in each sample was measured using an OxiSelect™ In Vitro ROS/RNS Assay Kit (Cell Biolabs, Inc., San Diego, CA, USA) with a modified protocol. Briefly, 150 μL of cytosolic fraction was added to a black 96-well plate and mixed with 25 μL of a catalyst, which helps to accelerate the oxidative reaction. After 5 min of incubation, 50 μL of the prepared dichlorodihydrofluorescin was added to all wells, and the oxidative reaction occurred fluorometrically for 45 min at 26 °C. Then, the fluorescence signal was measured at 530 nm excitation and 480 nm emission. ROS/RNS levels were measured using SpectraMax iD3 (Molecular Devices, San Jose, CA, USA). Blank sample fluorescence was subtracted from the sample value, and data were normalized by dividing the intensity with the weight of the sample.

### 2.4. Superoxide Dismutase (SOD) Activity

SOD activity was measured using a Superoxide Dismutase Activity Assay Kit (Abcam, Cambridge, UK) according to the manufacturer’s instructions. The volume of the solution used in the experiment was halved. Then, 10 μL of the cytosolic fraction was used without dilution. The assay utilized a water-soluble tetrazolium salt (WST-1) that produced a water-soluble formazan dye upon reduction with a superoxide anion. SOD catalyzes the dismutation of the superoxide anion into hydrogen peroxide and molecular oxygen, resulting in a decrease in the levels of WST-1, which has an absorbance at 450 nm. The inhibition of SOD activity was recorded using a SpectraMax iD3 (Molecular Devices, San Jose, CA, USA). The inhibition rate of the sample was normalized by dividing the absorbance unit by the weight of the sample.

### 2.5. Mitochondrial Oxidative Phosphorylation (OXPHOS) Complex Activity

Mitochondrial protein was quantified using the Bradford assay, and 500 ng of mitochondrial protein was used for measuring the levels of (OXPHOS) enzyme. The activities of OXPHOS complexes I, II, III, and IV were measured using spectrophotometry [24]. The change in the absorption of the reactant or product as an enzyme reaction was kinetically observed for 10 min with 10-sec intervals using a S-3100 UV-Vis spectrophotometer with a temperature controller (Scinco, Seoul, Korea). To assess the activity of complex I (NADH: ubiquinone oxidoreductase), reduced nicotinamide adenine dinucleotide (NADH), decylubiquinone (DUB), and 2,6-dichlorophenolindolphenol (DCPIP) were used as substrates. Mitochondria were added to the buffer of a Mitocheck Complex I Activity Assay Kit (Cayman Chemical, Ann Arbor, MI, USA) with 0.5 mg/L BSA, 0.2 mM NADH, 0.09 mM DCPIP, and 0.3 mM potassium cyanide (KCN) and were incubated for 10 min at 37 °C. Then, 0.05 mM of DUB was added to start the reaction. The decrease in absorbance at 600 nm, which correlated with a reduction in DCPIP levels, was monitored. To check the activity of complex II (succinate dehydrogenase), succinate, DCPIP, and DUB were used as substrates. Mitochondria were added to 500 mM potassium phosphate at pH 7.5 with 0.5 mg/L BSA, 0.3 mM KCN, 20 mM succinate, and 0.015% (wt/vol) DCPIP and were incubated for 10 min at 37 °C. Then, 0.05 mM of DUB was added to start the reaction. The decrease in absorbance at 600 nm, which correlated with a reduction in DCPIP levels, was monitored (*ɛ* = 0.0191 μM^−1^cm^−1^). To assess the activity of complex III (decylubiquinol (DUBH_2_) cytochrome *c* oxidoreductase), DUBH_2_ and ferric cytochrome *c* were used as substrates. Mitochondria were added to 500 mM potassium phosphate at pH 7.5 with 0.075 mM cytochrome *c*, 0.3 mM KCN, 0.1 mM ethylenediaminetetraacetic acid, and 2.5% (*v*/*v*) Tween 20 and were incubated for 10 min at 37 °C. Then, 0.05 mM of DUBH_2_ was added to start the reaction. The increase in absorbance at 550 nm that correlated with a reduction in the levels of cytochrome *c* was monitored (*ɛ* = 0.0185 μM^−1^cm^−1^). To assess the activity of complex IV (cytochrome *c* oxidase), reduced cytochrome *c* was used as a substrate. After incubating the solution containing 100 mM potassium phosphate at pH 7.0 and 0.06 mM reduced cytochrome *c* for 10 min at 37 °C, mitochondria were added to start the reaction. The decrease in absorbance at 550 nm, which correlated with the oxidation of cytochrome *c*, was monitored. All of the reagents were purchased from Sigma Aldrich (St. Louis, MO, USA). The *V_0_* as enzyme activities of the mitochondrial complexes were numerically calculated according to Equation (1), as follows:

Enzyme activity (*V_0,_* μM∙sec^−1^) = (ΔAbs/sec)/(extinction coefficient, *ɛ*) (1)

### 2.6. Statistical Analysis

Statistical analyses were performed using GraphPad Prism 8.0.1 software (GraphPad, San Diego, CA, USA). Statistical significance was assessed by the one-way analysis of variance, whereas multiple comparisons with the control group were performed using Dunnett’s test. The results are presented as the mean ± standard error of the mean (S.E.M.), and significant differences were established at *p* < 0.05.

## 3. Results

### 3.1. Decreased ROS/RNS Levels and SOD Activities Due to Low-Dose POP Exposure

**Figure 2:**
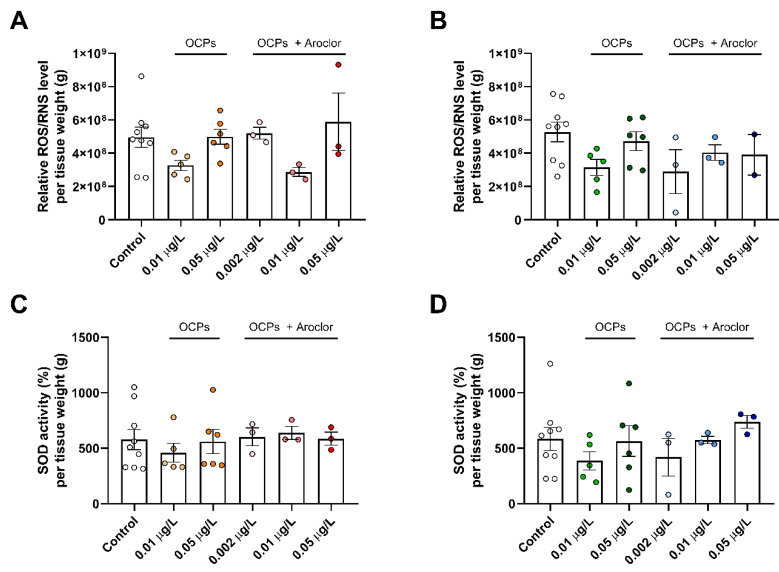
Relative reactive oxygen species (ROS)/reactive nitrogen species (RNS) levels of (**A**) females and (**B**) males and superoxide dismutase (SOD) activity of (**C**) females and (**D**) males. All data were normalized based on the weight of the tissue. Control groups are shown as white dots, 0.01 μg/L organochlorine pesticide (OCP)-exposed females as yellow dots, 0.05 μg/L OCP-exposed females as orange dots, 0.002 μg/L OCP-and-Aroclor-exposed females as light pink dots, 0.01 μg/L OCP-and-Aroclor-exposed females as pink dots, 0.05 μg/L OCP-and-Aroclor-exposed females as red dots, 0.01 μg/L OCP-exposed males as green dots, 0.05 μg/L OCP-exposed males as dark green dots, 0.002 μg/L OCP-and-Aroclor-exposed males as light blue dots, 0.01 μg/L OCP-and-Aroclor-exposed males as blue dots, and 0.05 μg/L OCP-and-Aroclor-exposed males as navy blue dots. Results represent the mean and S.E.M.

To determine the relationship between POP exposure and oxidative stress, the total levels of ROS and RNS and SOD activity were measured using cytosol from the tails of solitary OCP- or OCP-and-Aroclor-exposed zebrafish (Figure 2). Oxidative stress is a consequence of the increased generation of reactive radical species such as ROS and RNS, along with reduced physiological activity of antioxidant defenses against radial species [25].

In females, the total ROS/RNS levels upon OCP exposure were decreased under 0.01 μg/L exposure and then increased under 0.05 μg/L exposure, which is similar to the levels of ROS/RNS in the control group (Figure 2A). Regarding females exposed to OCPs with Aroclor, although the patterns of decrease and increase at 0.01 μg/L and 0.05 μg/L doses, respectively, were matched, 0.002 μg/L-dose-exposed zebrafish’s tails exhibited unchanged ROS/RNS levels compared to the controls. In males, the total ROS/RNS levels of OCP exposure exhibited a decrease under 0.01 μg/L exposure and then increased under 0.05 μg/L exposure, showing a similar pattern to that of females, but they showed a decrease in the total ROS/RNS levels at a relatively low concentration of OCPs with Aroclor, at 0.002 μg/L, which was a lower starting dose for a response than that observed for females (Figure 2B). This indicated that the dose required to change the ROS/RNS levels differed depending on the sex, and the response sensitivity of the ROS/RNS levels was higher in males than in females. Similarly, SOD activity decreased with the low dose of solitary OCPs and increased with the high dose in both females and males (Figure 2C,D). Regarding OCP and Aroclor exposure, females exhibited unchanged SOD activity in all exposed groups, whereas males exhibited decreased activity at low-dose exposure and then slightly increased activity at high doses compared to the control. This again indicated the sensitivity of the anti-oxidation system in response to POP exposure in males compared to females. Increased ROS generation and antioxidant depletion are common consequences of POP exposure. Previous studies with relative high doses of 200 μg/L POP exposure resulted in increases in ROS generation and depletion in the defense system [26,27]. The tendency towards increased oxidative stress in the previous results are contrast to the current result; nonetheless, the present results indicate a compensatory response of the ROS/RNS level upon very-low-dose POP exposure as and following a change in SOD activity that could attributed to the process of adaptation to the cellular conditions that prevents an immoderate anti-oxidizing action [28,29,30].

### 3.2. Differences in Mitochondrial Protein Levels upon Exposure to POPs in Females and Males

**Figure 3:**
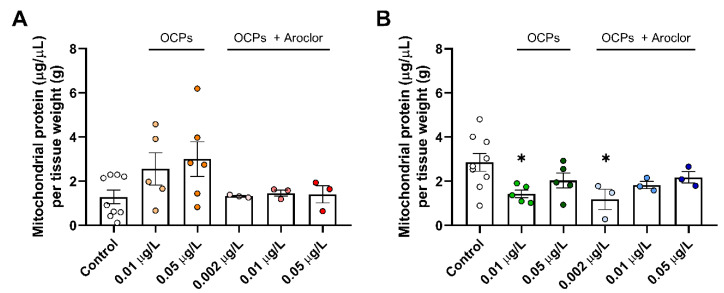
The amounts of mitochondrial proteins in the tails of persistent organic pollutant (POP)-exposed (**A**) female and (**B**) male zebrafish. Mitochondrial protein amounts were normalized according to the weight of the tissue. Control groups are shown as white dots, 0.01 μg/L organochlorine pesticide (OCP)-exposed females as yellow dots, 0.05 μg/L OCP-exposed females as orange dots, 0.002 μg/L OCP-and-Aroclor-exposed females as light pink dots, 0.01 μg/L OCP-and-Aroclor-exposed females as pink dots, 0.05 μg/L OCP-and-Aroclor-exposed females as red dots, 0.01 μg/L OCP-exposed males as green dots, 0.05 μg/L OCP-exposed males as dark green dots, 0.002 μg/L OCP-and-Aroclor-exposed males as light blue dots, 0.01 μg/L OCP-and-Aroclor-exposed males as blue dots, and 0.05 μg/L OCP-and-Aroclor-exposed males as navy blue dots. Results represent the mean and S.E.M. Statistical analysis was performed using GraphPad Prism 8.0.1 software. One-way analysis of variance was used to compare to the control. * *p* < 0.05 compared to the control.

The total mitochondrial protein levels were slightly changed upon exposure to POPs (Figure 3). Regarding solitary OCP exposure, females exhibited a compensatory increased amount of mitochondrial protein as the dose of toxins was increased up to 232% of controls under 0.05 μg/L exposure compared to the control group (Figure 3A). However, by exposing the fish to OCPs with Aroclor, the mitochondrial protein level was unchanged compared to that in the control group (Figure 3A). This may be due to either the minimal effect of the amount of mitochondria protein or a decrease after the compensation effect as an adaptive process to prevent the development of deleterious phenotypes upon exposure to solitary OCPs [31,32]. Contrarily, males exposed to both solitary OCPs and OCPs with Aroclor exhibited a changing decrease and increase pattern in the levels of mitochondrial protein (Figure 3B). Specifically, the amounts of mitochondrial protein decreased by 41% compared to controls under 0.002 μg/L exposure and increased to 76% of controls under 0.05 μg/L exposure. The initial decrease in mitochondrial protein before the compensatory increase could be a signal of mitochondrial compensation, and the starting dose for this signaling was increased in the group exposed to both OCPs and Aroclor compared to females. Based on the fact that male mitochondrial protein does not exhibit a compensatory increase compared to controls, it can be expected that the mitochondrial response upon POP exposure is more sensitive in females compared to males, which can be distinguished from the previous tendency towards a more sensitive response in males in terms of oxidative stress markers.

### 3.3. Inhibition of Mitochondrial Complex III Activity upon Exposure to POPs in Females

**Figure 4:**
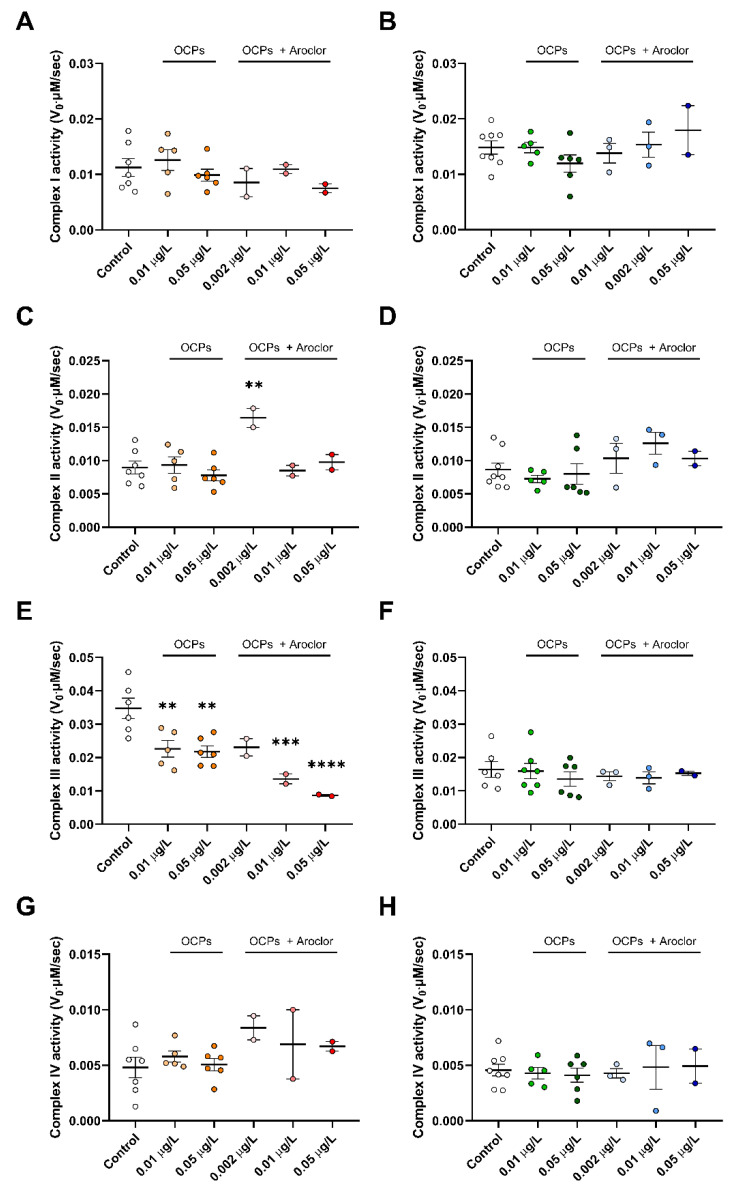
Mitochondrial complex I, II, III, and IV activities in the tails of female (**A**,**C**,**E**,**G**) and male (**B**,**D**,**F**,**H**) zebrafish exposed to POPs. Changes in absorbance were measured for 10 min at intervals of 10 s, and enzyme activities were calculated based on Equation (1). (**A**,**B**) Mitochondrial complex I activity. (**C**,**D**) Mitochondrial complex II activity. (**E**,**F**) Mitochondrial complex III activity. (**G**,**H**) Mitochondrial complex IV activity. Control groups are shown as white dots, 0.01 μg/L organochlorine pesticide (OCP)-exposed females as yellow dots, 0.05 μg/L OCP-exposed females as orange dots, 0.002 μg/L OCP-and-Aroclor-exposed females as light pink dots, 0.01 μg/L OCP-and-Aroclor-exposed females as pink dots, 0.05 μg/L OCP-and-Aroclor-exposed females as red dots, 0.01 μg/L OCP-exposed males as green dots, 0.05 μg/L OCP-exposed males as dark green dots, 0.002 μg/L OCP-and-Aroclor-exposed males as light blue dots, 0.01 μg/L OCP-and-Aroclor-exposed males as blue dots, and 0.05 μg/L OCP-and-Aroclor-exposed males as navy blue dots. Results are presented as the mean and S.E.M. Statistical analysis was performed using GraphPad Prism 8.0.1 software. One-way analysis of variance was used to compare to the control. ** *p* < 0.01, *** *p* < 0.001, and **** *p* < 0.0001 compared to the control.

Alterations in the function of the electron transport chain are known to be a major source of oxidative stress and are further responsible for the dysfunction of the overall cellular process [33,34]. Specifically, the dysfunction of the complex in the electron transport chain is linked to the onset of metabolic diseases [35,36]. Using the same amounts of mitochondrial protein, the activities of mitochondrial enzymes involved in the electron transport chain were measured (Figure 4). In the case of complex I, both females and males exhibited the same activity in the POP-exposed and control groups (Figure 4A,B). Likewise, complex II activity was also similar in all groups, except for the significantly increased activity in the 0.002 μg/L-dose-exposed females (Figure 4C,D). Complex IV activity was also similar in all groups, excluding the slightly increased activity in the 0.002 μg/L-dose-exposed females (Figure 4G,H). In contrast, complex III activity in the solitary OCP-exposed females was significantly inhibited; in the OCP-with-Aroclor-exposed groups, it was markedly more inhibited (Figure 4E,F). This represents the accelerating effect of Aroclor on the inhibitory action of OCPs on complex III activity. However, complex III activities in males were unchanged in all groups. In our previous study, we found that OCPs exert a competitive inhibitory action on complex III in isolated hepatic mitochondria [37]. According to these results, OCPs and the mixtures of OCPs with Aroclor are expected to have an effect similar to that of a specific inhibitor of complex III in the tail (muscular) mitochondria of females. The different lipophilicities of the tails of females and males might result in different accumulations of toxins and consequently incompatible inhibitory effects depending on the sex of zebrafish [38]. The increased effect of complexes II and IV found only in 0.002 μg/L-dose-exposed females can also be explained by the hypothesis that female individuals deposit more toxins in the tail, and thus, they are more affected by the accumulation of POPs than males.

## 4. Discussion

**Figure 5:**
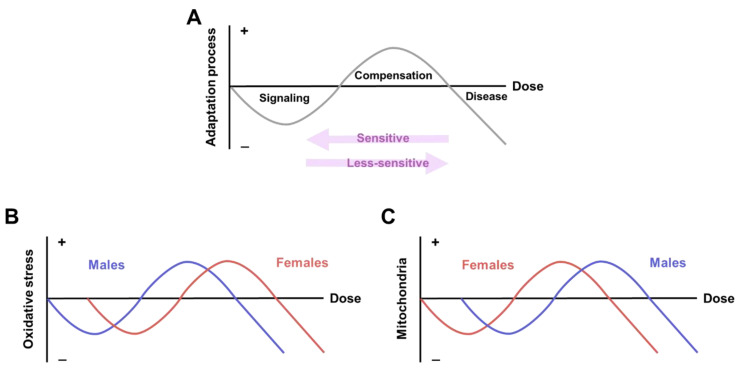
Metabolic adaptation and compensation process. (**A**) Process of metabolic adaptation. (**B**) Adaptation process of oxidative stress, which is sensitive in males. (**C**) Adaptation process of mitochondria, which is sensitive in females.

Early epidemiological studies revealed that POPs perturb metabolic health and are expected to have a synergetic effect when mixed. Here, we exposed zebrafish to two different types of POPs, OCPs and PCBs, and the results indicated the synergetic consequences of mixed exposure to the two subclasses; the toxicity of OCPs was found to be emphasized by the presence of Aroclor 1254. The results indicated different responses in oxidative stress and mitochondria upon exposure to OCPs or OCPs with Aroclor (Figure 5). Interestingly, the overall response tendency in oxidative stress and mitochondria was opposite in males and females. The oxidative stress markers, ROS/RNS levels, and SOD activities indicated a sensitive response upon exposure to OCPs with Aroclor in males compared to females (Figure 2 and Figure 5B). Specifically, males exhibited decreased ROS/RNS levels in the 0.002 μg/L OCP-and-Aroclor-exposed group and unchanged or increased SOD activities in all concentrations of the OCP-and-Aroclor-exposed groups, which is in contrast to the unchanged results observed in females. This result indicates that males are more sensitive (more alterations in oxidative stress marker levels) to OCPs and Aroclor exposure, whereas females exhibited less changes in marker levels. It should be noted that the total ROS/RNS levels were unchanged or decreased upon the POP exposure in both sexes. This would not represent the non-adverse effect of POPs but rather, the consequences of very-low-dose POP exposure, which has not been covered before. Moreover, previous studies have indicated that the reduction of the ROS/RNS level is not always followed by the improved function of the cell, since ROS functioning is one of the key regulatory factors for the signaling process [39,40,41].

However, markers of the mitochondrial protein amount and complex enzyme activity showed a sensitive response to OCP-and-Aroclor exposure in females compared to males (Figure 3,Figure 4,Figure 5C). Specifically, females exhibited compensatory increased mitochondrial protein levels in response to low-dose OCP exposure, whereas males exhibited decreased mitochondrial protein levels under the same dose. In previous studies, the compensatory response has occurred first in the sequence of signaling as a deleterious phenotype, followed by compensation as an ameliorated phenotype, and the failure of compensation has also occurred as a deleterious phenotype [31,32,42]. Accordingly, different levels of signaling and compensation are expected upon POP exposure, depending on sex; this is shown by the omitted signaling process for females in the present study. Moreover, females exhibited unchanged mitochondrial protein levels upon exposure to OCPs with Aroclor. This would be the middle step in compensation and failure, since females already show the compensatory response upon exposure to solitary OCPs; this again indicates the sensitive response of females upon exposure to Aroclor. Aroclor is a known endocrine-disrupting chemical that changes the level of estradiol [43,44]. In this context, the results of response sensitivity to OCPs with Aroclor in females could be caused by their having different endocrine systems to those of males, which would eventually result in contrasting responses to endocrine-disrupting chemicals. Although it is not clear whether the amount of OCPs and Aroclor deposited in the system is different depending on sex or not, this tendency toward a sensitive response in females is also demonstrated by the changes in mitochondrial enzyme activity.

In conclusion, the results of this study demonstrated that the effects of POP mixtures in the tails of zebrafish on oxidative stress markers and mitochondrial enzyme function differ according to sex and the addition of Aroclor, reflecting the real environmental conditions, with low-dose exposure to multiple toxins for a long time. We have elucidated the possible mechanistic processes, which involves a POP-induced imbalance in redox and hormone homeostasis and mitochondrial dysfunction, which are major causative factors for metabolic diseases such as type 2 diabetes. However, little is known about the different tendencies in relation to oxidative stress and oxidative phosphorylation markers between sexes, and about what specific metabolic changes are caused by exposure to POPs. The indeterminacy of the toxic effects of POPs in oxidative stress and metabolic changes needs to be investigated in further detail.

## Data Availability

The data that support the findings of this study are available from the corresponding authors, MC and SS, upon reasonable request.

## Abbreviations

Persistent organic pollutants, POPs; organochlorine pesticides, OCPs; polychlorinated biphenyls, PCBs; dichlorodiphenyltrichloroethane, 4,4′-DDT; hexachlorocyclohexane, beta-HCH; phosphate-buffered saline, PBS; bovine serum albumin, BSA; reactive oxygen species, ROS; reactive nitrogen species, RNS; superoxide dismutase, SOD; water-soluble tetrazolium salt, WST-1; oxidative phosphorylation, OXPHOS; reduced nicotinamide adenine dinucleotide, NADH; decylubiquinone, DUB; 2,6-dichlorophenolindolphenol, DCPIP; potassium cyanide, KCN; decylubiquinol, DUBH_2_.
